# A phase I/II study of irinotecan when added to 5-fluorouracil and leucovorin and pelvic radiation in locally advanced rectal cancer: a Colorectal Clinical Oncology Group Study

**DOI:** 10.1038/sj.bjc.6603570

**Published:** 2007-01-30

**Authors:** R Glynne-Jones, S Falk, T S Maughan, H M Meadows, D Sebag-Montefiore

**Affiliations:** 1 Mount Vernon Cancer Centre, Mount Vernon Hospital, Rickmansworth Road, Northwood, Middlesex HA6 2RN, UK; 2 Bristol Haematology and Oncology Centre, Bristol Royal Infirmary, Horefield Road, Bristol BS2 8ED, UK; 3 Department of Clinical Oncology and Palliative Medicine, School of Medicine, Cardiff University at Velindre Hospital, Whitchurch, Cardiff CF4 7XL, UK; 4 Cancer Research UK & UCL Cancer Trials Centre, 90 Tottenham Court Road, London, W1T 4TJ, UK; 5 Leeds Cancer Centre, Cookridge Hospital, Hospital Lane, Cookridge, West Yorkshire LS16 6QB, UK

**Keywords:** 5-fluorouracil, irinotecan, locally advanced rectal cancer, preoperative chemoradiation

## Abstract

The objective of this study was to evaluate the maximum tolerated dose (MTD) and recommended dose of irinotecan administered as a 5-day schedule synchronously with 5-fluorouracil (5FU), leucovorin (LV) and preoperative pelvic radiation (45 Gy) for primary borderline/unresectable, locally advanced rectal cancer. The study used escalating doses of intravenous irinotecan (6, 8, 10, 12, 14, 16, 18, and 20 mg m^−2^) administered on days 1–5 and 29–33 followed by low dose LV (20 mg m^−2^) and 5FU (350 mg m^−2^ over 1 h) in sequential cohorts. Preoperative pelvic radiotherapy using a three- or four-field technique and megavoltage photons comprised 45 Gy given in 25 fractions, 1.8 Gy per fraction. Surgery in the form of mesorectal excision was performed 6–10 weeks later. Histopathological examination of the resected specimen was performed according to techniques of Quirke, and compared with clinical staging. A distance of 1 mm or less between the peripheral extent of the tumour and the radial resection margin defined an involved circumferential resection margin (CRM). The MTD was determined as the dose causing more than a third of patients to have a dose-limiting toxicity (DLT) defined as specific grade 3 or 4 toxicities. Once the MTD was reached, a further 14 patients were treated at the dose level below the MTD. In total, 57 patients received irinotecan at the eight dose levels. The final cohort reached DLT after only four patients had been enrolled. The median age was 62 years (range 26–75), 37 male and 20 female subjects. The MTD of irinotecan in this schedule was 20 mg m^−2^ when three out of four patients experienced DLT. Dose limiting grade 3 or 4 diarrhoea was reported in seven out of 57 patients, three at the 20 mg m^−2^ dose level. Serious haematological toxicity (grade 3) was minimal and reported in only three patients; one grade 3 neutropaenia, one grade 4 neutropaenia and one grade 3 febrile neutropaenia and anaemia. Compliance was good with 93 and 89% of patients completing radiotherapy and chemotherapy, respectively. The remaining patients had only minor deviations from protocol therapy. Eight patients did not proceed to surgery, in six cases because they remained unresectable or had developed metastatic disease, one patient was unfit for surgery and one died as a result of complications from radiotherapy. Forty-nine patients underwent a potentially curative surgical resection. Histopathological examination of the resected specimen demonstrated pCR 12 out of 49 (24%) and 12 out of 57 (21%) overall. A histologically confirmed clear circumferential resection margin (CRM) was achieved in 39 out of 49 (80%) of those resected, and 39 out of 57 (68%) overall. In conclusion, MTD with this scheduled regimen of irinotecan is 20 mg m^−2^ (days 1–5 and 29–33). The acceptable toxicity and compliance at 18 mg m^−2^ recommend testing this dose in future phase III studies. The tumour downstaging and complete resection rates (negative CRM) are encouragingly high for this very locally advanced group.

A curative surgical resection remains the most important component of the modern multimodality management of rectal cancer. However, randomised controlled trials (Swedish Rectal Cancer Trial, 1997; Kapiteijn *et al*, 2001; Sauer *et al*, 2004) and two meta-analyses (Cammà *et al*, 2000; Colorectal Cancer Collaborative Group, 2001) have demonstrated that in clinically resectable rectal cancer, preoperative adjuvant radiation or chemoradiation can reduce the rate of locoregional recurrence, although its impact on overall survival is less clear.

Recent advances which have contributed to an improved outcome in rectal cancer include a more radical surgical technique incorporating mesorectal excision (TME) (Enker *et al*, 1995; Heald *et al*, 1998) and a more accurate histopathological examination of the resected specimen reporting the proximity of microscopic tumour to the circumferential resection margin (CRM) (Quirke *et al*, 1986). Individual surgical series, a population-based audit (Wibe *et al*, 2002) and evidence from a randomised controlled trial (Nagtegaal *et al*, 2002) all demonstrate lower rates of local recurrence following TME compared with the previous randomised trials that included a surgery alone arm. In addition, involvement of the circumferential resection margin is associated with a significantly increased risk of both local recurrence and also metastatic disease.

Preoperative chemoradiation (CRT) is increasingly used in patients with more locally advanced, borderline and unresectable rectal cancer, despite the fact that there are few trials in the modern era that have compared preoperative CRT with radiation alone (Frykholm *et al*, 2001). The aims of treatment are to reduce the extent of the primary tumour to allow macroscopic removal to take place, to treat potential microscopic disease close to or beyond the mesorectal fascia and if present also to treat micro-metastatic disease outside the radiation fields. Most current CRT schedules combine pelvic radiation with either continuous infusion of 5-fluorouracil (5FU), or 5FU modulated by LV. Mature results from the EORTC 22921 and FFCD 9203 trials of operable T3/T4 rectal cancer have recently demonstrated a convincing reduction in locoregional failure when a CRT regimen of 5FU and LV is added to 45 Gy of pelvic radiation over radiotherapy alone (Bosset *et al*, 2005a, 2005b; Gerard *et al*, 2005). As the EORTC 22921 and FFCD 9203 trials used daily 5FU LV based CRT schedules, at the time the present study was designed, it was a logical step to develop preoperative radiation schedules that incorporated the addition of irinotecan to the same regimen. The choice of the 5FU LV regimen required the 5-day schedule of irinotecan to be given in weeks 1 and 5 to ensure concomitant administration.

Preclinical studies have demonstrated irinotecan to be a potent radio-sensitising agent in human lung tumour xenografts (Tamura *et al*, 1997) and colorectal cancer (Wang *et al*, 1996; Omura *et al*, 1997; Chen *et al*, 1999), even under hypoxic conditions, which normally render tumours radio-resistant (Boscia *et al*, 1993). The mechanism of this interaction may reflect the attachment of the DNA-topoisomerase I adducts at sites of DNA single-strand breaks, or radiation-induced synchronization of the tumour cell population in the S phase of the cell cycle, where cells are more sensitive to irinotecan chemotherapy (Falk and Smith, 1992). Furthermore, the cytotoxic effects of camptothecins are highly schedule dependent. Both 5FU (Byfield *et al*, 1982) and irinotecan (Kirichenko *et al*, 1997) are radiation sensitisers, which appear both dose and schedule dependent according to preclinical models. In the preclinical setting, more frequent fractionated schedules appear more effective (Kirichenko and Rich, 1999). This hypothesis, to some extent, is logical if one considers that the half-life of SN38 (the metabolite of the camptothecins) is long, and a daily intravenous schedule can result in a relatively steady level. Radiation sensitisation occurs during or after irradiation (Omura *et al*, 1997). Hence, we have used a protracted low-dose schedule of irinotecan in the present study.

The rationale behind the use of these two drugs combined with radiotherapy lies in the evidence from phase III trials in metastatic colorectal cancer, which have demonstrated statistically significant improvements in overall response rate and median time to progression and overall survival when the topoisomerase-I inhibitor irinotecan is added to a combination of 5FU and LV (Douillard *et al*, 2000; Saltz *et al*, 2000). It is also recognised that 20–40% of rectal cancer patients continue to develop distant metastases and die despite the optimal use of TME and a very low risk of local recurrence (Kapiteijn *et al*, 2001; Sauer *et al*, 2004).

Also a recent review of phase II and III trials of preoperative chemoradiotherapy (Hartley *et al*, 2005) showed that on multivariate analysis only the mode of delivery of 5FU (infusion rather than bolus), the use of a second drug in addition to 5FU and the total radiation dose were associated with a higher rate of pathological complete response.

The present study aimed to evaluate the toxicity and efficacy of the addition of a 5-day schedule of irinotecan to a regimen of chemoradiation that uses 5FU and LV daily during the first and fifth weeks of pelvic radiation (45 Gy) as used in the EORTC 22921 and FFCD 9203 phase III trials (Bosset *et al*, 2005a, 2005b; Gerard *et al*, 2005).

## PATIENTS AND METHODS

### Study design

**Figure 1:**
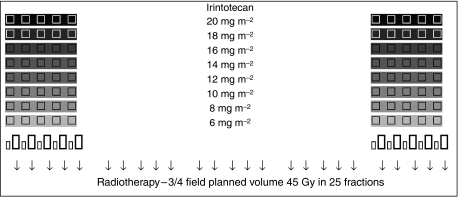
Chemoradiation schedule. □ Irintotecan at dose levels shown on days 1–5 and 29–33 prior to LV, 5FU; 
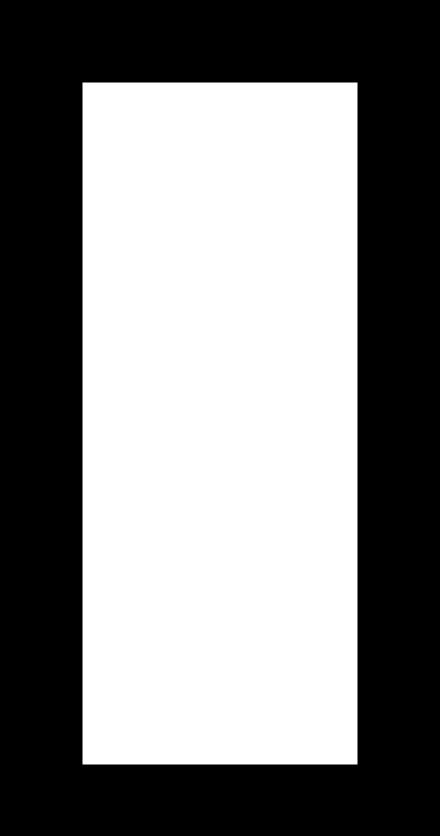
 Leucovorin 20 mg m^−2^ bolus days 1–5, 29–33; 
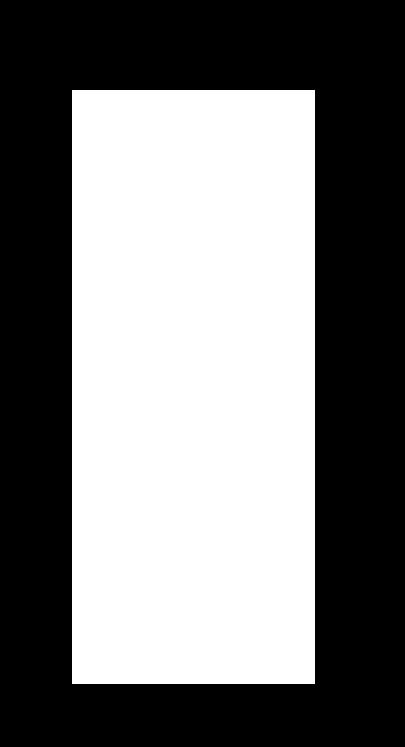
 5FU 350 mg m^−2^ 60 min infusion days 1–5, 29–33; ↓ radiotherapy 1.8 Gy per fraction.

This dose escalation study aimed to increase the dose of irinotecan given on days 1–5 and 29–33 in successive cohorts when added to a chemoradiation (CRT) regimen consisting of low-dose LV and a short infusion of 5FU administered concurrently with radiation until the maximum tumour dose was reached. All patients received radiotherapy (45 Gy) with LV (20 mg m^−2^) and 5FU (350 mg m^−2^ administered over 1 h) on days 1–5 and 29–33, see Figure 1. Escalating doses of 2 mg m^−2^ were given starting at 6 mg m^−2^ in three patients. Subsequent cohorts consisted of six patients. Irinotecan was administered in 100 ml normal saline or dextrose over 2 h given on days 1–5 and 29–33 (6, 8, 10, 12, 14, 16, 18 and 20 mg m^−2^) before the LV and 5FU. Dose-limiting toxicities were defined to include grade 3 or 4 diarrhoea, mucositis, grade 3 or 4 thrombocytopaenia and grade 4 neutropaenia associated with fever or lasting for more than 7 days according to the National Cancer Institute Canada common toxicity criteria revised 1994 definitions. Toxicity was recorded prospectively, weekly up to week 6 and then at week 10.

If Grade III or IV dose-limiting toxicity was observed in two or less patients in a cohort of six, then a further cohort were treated at the next dose level. The maximum tolerated dose (MTD) was defined if three or more of six patients experienced dose-limiting toxicity (DLT). Once the MTD was defined, the preceding dose level would then be expanded and a total of 20 patients were treated at the recommended dose.

The primary end points of the study were to determine the MTD of irinotecan. Secondary end points included acute toxicity, compliance with the planned doses of chemotherapy and radiotherapy, and efficacy. Efficacy was measured using the following: histopathological complete response rate, complete resection rate and local recurrence rate.

### Eligibility criteria

Eligibility criteria included histologically confirmed adenocarcinoma, WHO performance status 0–2 and no evidence of metastatic disease using chest X-ray and abdomino-pelvic CT. Acceptable haematological and renal function was required: neutrophils >1.5 × 10^9^ l^−1^, platelets ⩾100 × 10^9^ l^−1^ and serum creatinine <1.25 × the institutions upper limit of normal range.

Patients with locally advanced, biopsy proven carcinoma of the rectum were included either on the basis of fixity on digital rectal examination (DRE), T4 stage on pelvic CT or when MRI demonstrated a high risk of involvement of the circumferential resection margin. The method used to define locally advanced disease, distance of inferior tumour border to the anal verge (cm) and intended surgical procedure were also recorded.

Patients were excluded from the study because of prior chemotherapy or pelvic radiation, lack of efficient contraception or pregnancy and bowel or cardiac conditions that would deter the safe delivery of 5FU. Patients having six episodes of stool per day or who were incontinent of faeces were also excluded.

### Pelvic radiation

Using information from clinical examination and pelvic MRI, the gross tumour volume (GTV) was defined using a CT planning scan. Alternatively, orthogonal film simulation was performed with opacification of the small bowel using barium sulphate 300 ml with gastrograffin 20 ml. To derive a planning target volume (PTV) margins were added to the GTV according to the radiation planning diagrams (3 cm laterally and inferiorly, 2 cm anteriorly and superiorly) included in the protocol with the exception of the posterior border, which was always located on the most posterior aspect of the bony sacrum. Patients were treated prone with a full bladder using either a three- or four-field technique. A total dose of 45 Gy was delivered to the International Commission on Radiation Units intersection point using 25 daily fractions of 1.8 Gy.

### Surgery and histopathology

Surgery was recommended to take place 6–10 weeks after completion of chemoradiation. Histopathological examination of the resected specimen was performed according the technique described by Birbeck and Quirke (1999). The circumferential resection margin is considered involved, if microscopic tumour (due to any cause) is present at or less than 1 mm from the circumferential or radial resection margin.

### Assessment during and after treatment

Full blood count, urea creatinine and electrolytes and liver function tests and acute toxicity scores were assessed prospectively on weeks 1–6 and 10. On completion of CRT, follow-up appointments were given at 3, 6 12, 24 and 36 months to assess tumour recurrence and late toxicity.

For the purposes of this study, local recurrence has been defined in patients who have had a complete macroscopic resection as evidence of either an intraluminal or extraluminal mass below the sacral promontory and biopsy proven adenocarcinoma or CEA abnormal with or without the presence of metastases. Radiological evidence of interval enlargement of the mass (minimum interval of 6 weeks) was required if based on CT scans alone (biopsy negative and CEA normal).

## RESULTS

Between June 2000 and May 2005, a total of 59 patients were recruited from four centres. Two patients were excluded; one patient stopped chemotherapy owing to 5FU-induced chest pain, and the second patient represented a protocol deviation because they received bolus 5FU instead of infusional 5FU. The characteristics of the 57 eligible patients are summarised in Table 1.

### Protocol compliance

Protocol compliance was good, although one patient progressed during treatment and stopped their radiotherapy and chemotherapy early. Radiotherapy was minimally reduced (one fraction not given) in three patients owing to side effects (pain, illness and diarrhoea), all other patients received the full radiotherapy dose. Only five patients failed to receive all planned chemotherapy, which was reduced in four cases due to toxicity; pain (one), diarrhoea (two) and weight loss (one). The remaining patient fainted prior to treatment and that day's chemotherapy treatment was omitted.

The median field size, in all patients, for the posterior/anterior field was 15.2 cm (range 9.8–20.5) length × 14.0 cm (range 9.9–18.1) PA width, and for the lateral/oblique fields 15.1 cm (range 9.8–20.5) height × 13.0 cm (range 9.9–17.7) AP field size on lateral. Forty-nine out of 57 patients proceeded to have radical surgery, with a median interval to surgery of 10 weeks (IQR 7–12 weeks).

### Acute toxicity

The acute grade 3/4 toxicity experienced is summarised for the eight dose levels and the subsequent patients treated in the expanded cohort at 18 mg m^−2^ in Table 2. During the dose escalation phase, DLT was seen in three out of four patients at 20 mg m^−2^. In view of this toxicity, the decision was made to stop recruitment to this dose level and to expand the18 mg m^−2^ dose level to the planned total of 20 patients.

The most common toxicity was grade 3 diarrhoea, this occurred in seven patients. Haematological toxicity was uncommon with only three patients affected; grade 3 neutropaenia (14 mg m^−2^), grade 4 neutropaenia (18 mg m^−2^) and grade 3 febrile neutropaenia and grade 3 anaemia (8 mg m^−2^). One patient died 2 weeks after chemoradiation of complications following radiation ileitis.

### Response to treatment

Tumour downstaging was defined by comparing clinical TN stage prior to treatment (as determined by pelvic MRI) with histopathological stage post surgery. Chemoradiation therapy achieved downstaging in 20 out of 49 (41%) of patients. The pathological stages at surgery were ypT0N0=12, ypT1NO=1, ypT0N1=1, ypT1N1=0, ypT2N0=7, ypT2N1=2, ypT3N0=14, ypT3N1=4, ypT3N2=2 and ypT4N0=2, ypT4N1=3, ypT4N2=1. It can be seen that CRT achieved a complete pathological response in 12 out of 49 (25%) of cases who proceeded to surgery (Table 3) and 12 out of 57 (21%) of the whole group. Histopathology also demonstrated microscopic disease only (Tmic) in two out of 49 (4%) patients, pCR+ Tmic in 14 out of 49 (29%), pT0-2 in 20 out of 49 (41%) and histologically confirmed (>1 mm) clear circumferential resection margins (CRM) in 41 out of 49 (84%) of those resected, and 39 out of 57 (68%) overall.

### Surgery

**Figure 2:**
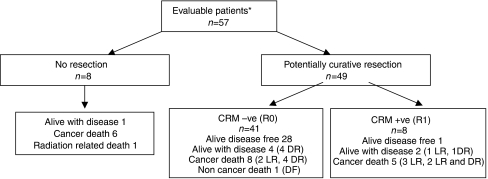
Patient outcome. ^*^Two unassessable patients not included; one patient discontinued chemotherapy owing to chest pain and the other had bolus 5FU on day 1. LR=locoregional recurrence, DR=distant recurrence, DF=disease free.

Of the 57 evaluable patients, eight did not proceed to surgery, in six cases because they remained unresectable (two) or had metastatic disease (four), one patient was unfit for surgery and one died as a result of complications from radiotherapy (Figure 2). All patients had a preliminary clinical assessment by their surgeon at entry and were categorised on the basis of height of the cancer from the anal verge as requiring an abdomino-perineal resection (APER), anterior resection (AR) or pelvic clearance (PC).

Of the 49 radical surgical procedures, 24 had an APER, 20 patients an AR, one patient had a low Hartmann's operation. Three female patients required APER and total abdominal hysterectomy and bilateral salpingo-oopherectomy and a male patient had an AR and removal of the bladder and prostate. There were no unexpected postoperative complications or deaths.

### Local recurrence and survival

**Figure 3:**
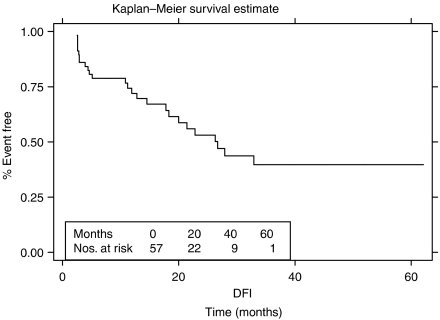
Disease-free survival – all patients.

The median follow-up is 16 months (range 0–60 months) for all patients, 23 patients (40%) have follow-up of 2 years or more. Thirty-seven of the 57 patients remain alive at the time of writing. Of the 41 patients having complete surgical clearance of tumour (CRM negative), 11 relapsed (one at local and distant sites and 10 distant sites only), of which seven subsequently died of disease (Figure 2 and Table 4). Of the eight incomplete resections (CRM positive), five patients have died, three are alive; two with disease and one currently remains disease free. All but one of the eight patients who were not resected have died, the remaining patient is alive with disease. The disease-free survival (DFS) for the whole group at 3 years is 40% (95% CI 24–55%) (see Figure 3) and 54% (95% CI 32–72%) for those having had a complete resection.

Of the 20 patients who were downstaged to T0-2 NO, 15 remain alive and disease free, two are alive with distant metastases and three have died of distant disease. Of the 29 patients that were not downstaged (T3/4 NO/1 or T0-2 N1 disease), 10 died due to disease, 18 are alive; four with disease and 14 disease free. The remaining patient died of a myocardial infarction but was disease free at the time of death.

### Late effects

Severe late effects were uncommon despite the combination of irinotecan and radiotherapy with one patient reporting Grade 3 tenesmus at assessment 12 months after treatment, which later improved to grade 1/2 and had resolved by 4 years.

## DISCUSSION

The primary aim of our current treatment strategy in rectal cancer is to ensure long-term local control, by achieving a curative resection with a clear circumferential resection margin (>1 mm). However, in many patients with more locally advanced disease, the tumours often remain fixed and unresectable following CRT, and others fail to achieve a histologically curative resection despite neoadjuvant chemoradiation (Mawdsley *et al*, 2005). A number of phase II studies using 5FU have reported high rates of PCR and R0 resection even in advanced low rectal cancers. However, the incidence of metastases remains unacceptably high for this group of patients, and does not appear to be influenced by the addition of 5FU over radiation alone (Bosset *et al*, 2005a, 2005b; Gerard *et al*, 2005). Intensification of the neoadjuvant strategy has, therefore, been tested in terms of the addition of further cytotoxic agents such as oxaliplatin and irinotecan to standard 5FU-based chemoradiation in an attempt to improve efficacy of downstaging, improve resectability and eradicate potential micrometastases more effectively. Irinotecan appears suitable for inclusion in a neoadjuvant chemoradiation regimen based on the higher response rates observed in randomised trials in metastatic disease (Douillard *et al*, 2000; Saltz *et al*, 2000).

The MTD of a scheduled regimen of irinotecan combined with low-dose LV and a 60 min infusion of 5FU with 45 Gy of pelvic radiotherapy (using relatively large field sizes) given preoperatively in patients with locally advanced rectal cancer has been defined in the present study as 20 mg m^−2^, days 1–5 and 29–33. The recommended dose in this setting is 18 mg m^−2^. This overall total dose of irinotecan (180 mg m^−2^) is similar to that achieved by others using a schedule of 40–60 mg m^−2^ weekly during radiotherapy (Levine *et al*, 2003; Mehta *et al*, 2003).

Compliance with treatment (93% radiotherapy and 89% chemotherapy) in the present study compares favourably with the EORTC 22921 study where compliance to RT was 96% and to chemotherapy was only 71% (Bosset *et al*, 2004). In addition, the rate of grade 3/4 diarrhoea in this study of 12% is lower than other similar studies with a weekly regimen of irinotecan, which range from 30 to 40% (Mitchell *et al*, 2001; Mehta *et al*, 2003; Klautke *et al*, 2005).

The eligibility criteria used in this study define a relatively homogeneous group of advanced borderline/unresectable rectal cancer. A total of 23 out of 57 (40%) patients had tumours, which were clinically fixed. The PCR rate of 21% in the present study is high compared to CRT regimens, which use single agent 5FU or capecitabine, and is similar to other reported irinotecan CRT regimens (see Table 5).

The histologically confirmed RO (CRM −ve) resection rate of 83% for those resected and 68% overall is acceptable for studies in this group of borderline unresectable rectal cancers (Wheeler *et al*, 2004; Mawdsley *et al*, 2005; Sebag-Montefiore *et al*, 2005). In the present study, only two patients had disease, which remained fixed and unresectable following CTRT, and only three patients had partial pelvic clearances. In contrast, the German CAO/ARO/AIO 94 study (Rodel *et al*, 2003) reported eight out of 31 patients who underwent exenteration or resection of adjacent organs.

Enthusiasm for preoperative chemoradiation in the management of rectal cancer is increasing. The German CAO/ARO/AIO 94 study protocol has convincingly shown improved locoregional control and reductions in acute and late toxicity with preoperative chemoradiation (Sauer *et al*, 2004) *versus* postoperative combined modality treatment for stage II/III resectable rectal cancer.

There is considerable enthusiasm for integrating irinotecan into preoperative combined regimens in rectal cancer, and there have been several reported phase I and II studies (Levine *et al*, 2003; Mehta *et al*, 2003; Mitchell *et al*, 2003; Klautke *et al*, 2004). Most investigators have explored weekly schedules of irinotecan with infusional 5FU in combination with pelvic radiotherapy (Mitchell *et al*, 2001; Navarro *et al*, 2006).

The first study was performed by Mitchell in 2001; as with other studies, high initial PCR rates have not been confirmed with a larger number of patients (Mitchell *et al*, 2003). An update of this study (Kim *et al*, 2005) suggests that the pCR rate may be much higher for small tumours< 5 cm. They showed that 11 out of 28 (39%) of small tumours achieved PCR, compared to only five out of 30 (17%) of tumours larger than 5 cm. Final recommendations from the results of this study were to use 250 mg m^−2^ per day of 5FU by continuous infusion, and 50 mg m^−2^ weekly of irinotecan combined with a radiation dose of 54 Gy. This regimen has been formally compared with a twice-daily hyperfractionated regimen in a randomised phase II study under aegis of the RTOG (RTOG R-0012). UK investigators have explored similar schedules (Levine *et al*, 2003) and produced a recommended dose of 5FU by continuous infusion 200 mg m^−2^ and irinotecan 60 mg m^−2^ weekly with 45 Gy of radiotherapy.

Further investigators have also combined capecitabine and irinotecan (Kennedy *et al*, 2004; Becerra *et al*, 2005; Gollins *et al*, 2005; Willeke *et al*, 2005). While preliminary, the current findings indicate that combinations of capecitabine and weekly irinotecan are feasible in this setting. Capecitabine doses can be up to 825 mg m^−2^ twice daily continuously, while irinotecan can be given up to 60 mg m^−2^, if only four infusions are administered.

Currently, there is a further ongoing randomised phase II study in the preoperative setting under the aegis of the RTOG (RTOG-0247), which compares a combination of irinotecan and capecitabine with a combination of oxaliplatin and capecitabine and higher doses of radiotherapy (50.4 Gy) than in the present study. Similarly, Klautke *et al* (2005) also explored 50.4 Gy with continuous infusion 250 mg m^−2^ per day and irinotecan 40 mg m^−2^ weekly, and described an incidence of grade 3 diarrhoea of 35%. This level of toxicity is probably unacceptable for a multicentre phase III study despite a 26% pathological complete response. An even higher complete response rate has been reported from Mehta *et al* where again patients were treated with 50.4 Gy conformal radiotherapy and continuous infusion 5FU 200 mg m^−2^ per day and 50 mg m^−2^ weekly × 4 of irinotecan. This schedule also provoked a high incidence of grade 3 diarrhoea (28%), although there was an impressive 37% pCR rate.

In the preoperative treatment of rectal cancer, it remains to be seen whether the rate of achieving a PCR is an independent prognostic factor for survival. Few studies report long-term local control, late toxicity or the subsequent incidence of metastatic disease. We are continuing to follow this group of patients and hope to provide better information on these issues in the future. Nevertheless, the high levels of pathological complete response with irinotecan need to be confirmed in randomised studies, and the present 5-day schedule would be interesting to assess in combination with capecitabine in phase III randomised trials.

We have yet to determine the optimal irinotecan-based preoperative chemoradiation regimen in rectal cancer. Phase I/II trials confirm that the combination is feasible and highly effective, but many of the studies have shown high levels of grade 3 toxicity – in terms of diarrhoea, see Table 5.

The present study has a number of important differences from the previously described studies (see Table 5). Firstly, the total dose of radiation was fixed at 45 Gy, a total dose that is 10% lower than many of the other studies. This dose might be expected to be associated with a lower incidence of late complications, and also a lower rate of pathological complete response (Hartley *et al*, 2005). Secondly, this study formally determined the MTD and recommended dose of irinotecan when added to a validated CRT fluoropyrimidine schedule used in a phase III trial (Bosset *et al*, 2005a, 2005b). Finally, this study reports outcome data that is based on the circumferential margin status. Now that there is clear evidence that locoregional control is improved by the addition of the 5FU/LV regimen used in this study to 45 Gy of pelvic radiation (Bosset *et al*, 2005a, 2005b; Gerard *et al*, 2005), there is a strong rationale for future trials to establish the benefit of the addition of irinotecan.

These data suggest that combination radiochemotherapy leads to improved early histopathological outcome measures. This has the potential to translate into improved long-term outcomes in rectal cancer, both in terms of quality of life and overall survival and will be tested in current and future phase III trials.

## CONCLUSION

The maximum tolerated dose of this scheduled regimen is confirmed as 20 mg m^−2^, and the recommended dose of irinotecan in BURC is 18 mg m^−2^given on days 1–5 and 29–33 when added to 5FU/LV and 45 Gy of radiation preoperatively. We are continuing to follow this group of patients and hope to provide better information on long-term local control, late toxicity or the subsequent incidence of metastatic disease in the future.

## Figures and Tables

**Table 1:** Patient and tumour characteristics

**Characteristic**	***n*=57**
Median age (range)	62 (26–75)
Male : female	37 : 20
WHO status 0 : 1 : 2	39 : 16 : 2
	
*Site of tumour*	
Upper : mid : lower	8 : 18 : 31
	
*Local extent*	
Fixed/unresectable	23
Locally advanced on MRI	33^a^
Increase chance of sphincter preservation	1

a Includes three patients with partially fixed unresctable disease also.

WHO=World Health Organisation; MRI=magnetic resonance imaging.

**Table 2:** Acute toxicity

**Irintotecan dose (mg m^−2^)**	**6**	**8**	**10**	**12**	**14**	**16**	**18^a^**	**20**	**All**
No. of patients	3	6	6	6	6	6	6/14	4	57
*Patients with DLT*	0	0	2	0	1	0	1/−	3	7
Gd 3 diarrhoea	—	—	2	—	1	—	1/−	3	7
Gd 3 neutropaenia	—	—	—	—	1	—	−/−	—	1
Gd 4 neutropaenia	—	—	—	—	—	—	−/1	—	1
Gd 3 febrile neut	—	1	—	—	—	—	−/−	—	1
Gd3 anaemia	—	1	—	—	—	—	−/−	—	1
Gd 3 nausea	—	—	—	—	1	—	1/−	—	2
Gd 3 vomiting	—	—	1	—	1	—	1/−	—	3
Gd 3 radiation derm	—	—	1	—	—	—	−/−	—	1
Gd 4 rash/desq	—	—	—	—	—	—	1/−	—	1
Gd 3 pain	—	—	—	—	1	—	−/1	—	2
Gd 4 pain	—	—	—	—	—	—	1/−	—	1
Gd 3 constipation	—	—	—	—	—	—	−/1	—	1

a The figures in this column indicate the numbers in the initial six patients treated at this dose level followed by the numbers in the expanded group once the MDT was determined.

DLT=dose-limiting toxicity.

**Table 3:** Pathological response

**Irintotecan dose (mg m^−2^)**	**6**	**8**	**10**	**12**	**14**	**16**	**18^a^**	**20**	**All (%)**
Number of patients	3	6	6	6	6	6	6/14	4	57
*Number operated*	3	5	6	5	5	5	5/12	3	49/57 (86%)
pCR	1	0	1	1	1	1	3/3	1	12/49 (25%)
Tmic	0	0	0	0	0	0	1/0	1	2/49 (4%)
RO (–ve CRM)^b^	3	3	6	4	5	4	5/8	3	41/49 (84%)
pT0-2 pN0	2	0	3	1	2	1	4/5	2	20/49 (41%)

a The figures in this column indicate the numbers in the initial six patients treated at this dose level followed by the numbers in the expanded group once the MDT was determined.

b −ve CRM=tumour clearance of more than 1 mm from circumferential resection margin; pCR=histopathological complete response; Tmic=microscopic disease only detected in surgical specimen; CRM=circumferential resection margin.

**Table 4:** Pattern of recurrence following radical resection

**Irintotecan dose (mg m^−2^)**	**6**	**8**	**10**	**12**	**14**	**16**	**18**	**20**	**All**
Number of patients	3	5	6	5	5	5	5/12	3	49
Any disease	2	1	3	3	3	1	2/4	0	19 (39%)
Local recurrence	1	1	0	2	0	1	0/3	0	8 (16%)
Distant metastases	2	1	3	1	3	1	2/1	0	14 (29%)
Cancer death	2	1	2	3	2	1	0/2	0	13 (27%)
Other death	0	0	0	0	0	1^a^	0/0	0	1 (2%)

a Cardiac death, no cancer present at time of death.

**Table 5:** Preoperative chemoradiation schedules using irinotecan

**Study/year**	**No. of patients**	**Fluoro-pyrimidine**	**Irinotecan schedule**	**RT dose (Gy)**	**G3/4 diarrhoea (%)**	**PCR (%)**
Levine/2003	12	PVI : 5FU 200 mg m^−2^, daily over 5 weeks	60 mg m^−2^ weekly × 4	45.0	25	—
Mehta/2003	32	PVI : 5FU 200 mg m^−2^, days 1–33	50 mg m^−2^ weekly × 4	50.4	28	38
Mitchell/2003	67	PVI : 5FU 225 mg m^−2^, 5 days a week	50 mg m^−2^ weekly × 4	54.0	—	25
Klautke/2005	37	PVI : 5-FU 250 mg m^−2^, days 1–43	40 mg weekly × 6	50.4	32	22
Navarro/2006	74	PVI : 5FU 225 mg m^−2^, 5 days a week	50 mg m^−2^ weekly × 5	45.0	14	14
Descartes/2006	57	5FU 350 mg m^−2^, LV (20 mg m^−2^, days 1–5 and 29–33	18 mg m^−2^ days 1–5 and 29–33	45.0	12	25
Hofheinz/2005	19	Capecitabine 500 mg m^−2^, b.i.d., days 1–38	50 mg m^−2^ weekly × 5	50.4	16	21
Gollins/2006	40	Capecitabine 650 mg m^−2^, b.i.d., days 1–33	60 mg m^−2^ weekly × 4	45.0	28	25
Klautke/2006a (BJC)	28	Capecitabine 750 mg m^−2^, b.i.d., days 1–43	40 mg weekly × 6	50.4+5.4	39	14
Klautke/2006b (ASCO)	20	Capecitabine 750 mg m^−2^, b.i.d., days 1–14, 22–35	50 mg weekly × 4	50.4+5.4	10	0
Klautke/2006b (ASCO)	11	Capecitabine 750 mg m^−2^, b.i.d., days 1–14, 22–35	60 mg weekly × 4	50.4+5.4	9	33
Mitchell/2006	16	Capecitabine, MTD not yet reached	50 mg m^−2^ weekly × 4	50.4 or 54	—	23

PVI=protracted venous infusion; RT=radiotherapy; PCR=polymerese chain reaction; MTD=maximum tolerated dose; 5FU=5-fluorouracil.
